# “*We are doing it together*”; The integral role of caregivers in a patients’ transition home from the medicine unit

**DOI:** 10.1371/journal.pone.0197831

**Published:** 2018-05-24

**Authors:** Shoshana Hahn-Goldberg, Lianne Jeffs, Amy Troup, Rasha Kubba, Karen Okrainec

**Affiliations:** 1 OpenLab, University Health Network, Toronto, Ontario, Canada; 2 School of Health Policy and Management, Yok University, Toronto, Ontario, Canada; 3 Keenan Research Centre of the Li Ka Shing Knowledge Institute, St. Michael’s Hospital, Toronto, Ontario, Canada; 4 University Health Network, Toronto, Ontario, Canada; 5 Department of Medicine, University of Toronto, Toronto, Ontario, Canada; Radboud University Nijmegen, NETHERLANDS

## Abstract

**Background:**

An admission to hospital for acute illness can be difficult for patients and lead to high levels of anxiety. Patients are given a lot of information throughout their hospital stay and instructions at discharge to follow when they get home. For complex medical patients, the ability to retain, understand, and adhere to these instructions is a critical marker of a successful transition. This study was undertaken to explore factors impacting the ability of patients to understand and adhere to instructions.

**Methods:**

A qualitative design of interviews with patients and caregivers was used. Participants were adult patients and caregivers with congestive heart failure, chronic obstructive pulmonary disease, or community-acquired pneumonia being discharged home from three academic acute care hospitals in Ontario, Canada. Semi structured interviews were conducted with participants within one week following their discharge from hospital. Interviews were audiotaped and transcribed. Five independent researchers participated in an iterative process of coding, reviewing, and analyzing the interviews using direct content analysis.

**Results:**

In total, 27 participants completed qualitative interviews. Analysis revealed the role of the caregiver to be critical in its relation to the ability of patients to understand and adhere to discharge instructions. Within the topic of caregiving, we draw on three areas of insight: The first clarified how caregivers support patients after they are discharged home from the hospital. The second highlighted how caregiver involvement impacts patient understanding and adherence to discharge instructions. The third revealed system factors that influence a caregiver’s involvement when receiving discharge instructions.

**Conclusion:**

Caregivers play an important role in the transition of a complex medical patient by impacting a patient’s ability to understand and adhere to their discharge instructions. The themes identified in this paper highlight opportunities for healthcare providers and institutions to effectively involve caregivers during transitions from acute care hospitals to home.

## Introduction

An admission to hospital for acute illness is a difficult time for patients marked with high levels of anxiety. Studies have consistently found high rates of poor retention and recall of discharge instructions by patients in the period following discharge [1–5]. Moreover, studies have documented that improved patient education through clarity and consistency of discharge instructions can contribute to improved adherence [6, 7]. Conversely, poor adherence can lead to adverse events and potentially avoidable outpatient or emergency department visits [8–10]. The recent shift to quality-based funding in healthcare incentivizes a decreased length of stay in acute-care and early follow-up following hospital discharge [11], which inadvertently puts more responsibility on patients to adhere to discharge instructions. A recent systematic review demonstrated that while several transitional care bundles target patient education as a means to improve retention of discharge instructions, the level of patient engagement varied widely and was often absent [12]. Moreover very few studies studied tools which improved adherence to instructions [12]. Identifying factors which aid in understanding and adherence to discharge instructions following admission to hospital is therefore paramount.

To address this gap in evidence, a qualitative study was undertaken to explore factors that impact the ability of patients to understand and adhere to discharge instructions. This paper presents insights from one of the largest themes that emerged, caregiver involvement during transitions home. This study is part of a larger mixed-method evaluation of the Patient-Oriented Discharge Summary (PODS), a discharge instruction tool that was co-created with patients to improve patient engagement while providing discharge instructions [13, 14].

## Methods

This study used a qualitative design methodology and was conducted in Canada. Ethics approval for the study, including all data collection materials and the consent process, was obtained at the University Health Network Research Ethics Board in January 2016 and at Thunder Bay Regional Health Sciences Centre in April 2016.

Participants in this study were all enrolled in an ongoing, double blinded, randomized controlled trial evaluating the impact of PODS [15]. Participants from both the intervention and control arm of the study were included. Patients, and their caregivers, admitted to acute medical care wards at three Ontario hospitals, between March and November 2016, were included in the study; Toronto General Hospital, Toronto Western Hospital, and Thunder Bay Regional Health Sciences Centre. Eligibility criteria for patients included the following: Greater than 18 years of age, being discharged home or to a retirement home, and having a diagnosis of Congestive Heart Failure, Chronic Obstructive Pulmonary Disease, or Community Acquired Pneumonia aligned with ministry directives to improve care transitions for patients with quality-based procedures [11].

All patients who met inclusion criteria were approached by a member of the research team while still in hospital to provide a study overview and obtain written, informed consent. Patients and caregivers were given the option of choosing which one of them would participate in the follow up with the study team. If they opted for the caregiver to participate, written, informed consent was obtained for the caregiver as well. Within one week following the patient’s discharge, a research assistant phoned patients or their family members to collect quantitative information related to their discharge experience as well as conduct optional qualitative interviews. All participants enrolled in the trial were given the option of participating in an interview. If the participant did not speak English, an over-the-phone professional interpreter was used. Participants were contacted individually by phone, rather than in-person, due to the feasibility of contacting people across the province within one-week post-discharge, an element of the protocol of the larger, mixed-methods trial.

An open-ended interview guide was used to elicit experiences associated with understanding and adherence to discharge instructions. This guide was designed by the research team based on a review of the literature and previous experience implementing a patient oriented discharge summary [13]. Separate guides were created for patients and caregivers (Table 1). The main difference between the guides was that the caregivers were also asked questions related to their role as caregivers. Interviews lasted between 12 and 39 minutes with an average length of 23 minutes. In addition to open responses of participants during the interviews, the researchers also collected descriptive information about the patients and quantitative data about their experience. Patient demographic information was collected from the patient at the time of consent and included admission diagnosis; age; sex; length of stay; presence of a self-reported physical or sensory disability; a measure of health literacy [16]; the presence of a language barrier, collected using a combination of measures including self-reported limited proficiency in English and language preference as indicated on the electronic patient record. Information about the patient and caregiver relationship collected included whether the patient lived with their caregiver and whether they relied on their caregiver for various tasks. At the follow up call, participants were asked to provide a subjective rating describing engagement during discharge teaching, a rating of their satisfaction, ratings of how well they understood various elements of the discharge teaching, and answer yes or no to whether they received publically-funded home care services and whether they had discussions with their care team in hospital about if they would have the help they needed at home.

**Table 1:** Patient and caregiver interview topics and questions.

Topic	Sample patient questions	Sample caregiver questions
Understanding discharge instructions	Can you tell me about your understanding of the discharge instructions you received in hospital? Prompts: Who gave you the instructions? Did you feel involved? Did you understand what you were being told to do?	Can you tell me about your understanding of the discharge instructions your relative received in hospital? Prompts: Were you there when discharge instructions were given? Did you understand what your relative was being told to do? Did you feel involved?
Using discharge instructions	How did you use your discharge instructions? Was there anything you particularly liked about your instructions? Was there anything you didn’t like about your instructions?	How did you use the discharge instructions? Did you use them to guide your relative’s care?
Social factors effect on adherence to instructions	How would you describe the effect your [insert relevant factor such as gender, language, race/ethnicity, socioeconomic status, disability] had on your ability to use and adhere to your discharge instructions?	Did the fact that your relative/you are [insert relevant factor- relevant for language barriers, disability only] affect the amount of help your relative needed from you?
Other resources accessed post-discharge	Can you tell me about any other programs that you are involved in to manage your health needs now?	Can you tell me about any other programs that your relative is involved in to manage their health needs now (care pathways, virtual ward, etc)?
Caregiving (question only for caregiver interviews)	Not applicable	How long have you been caring for [your relative]? What has that been like for you? How would you describe your role prior to and after (your relative) was in hospital?
Open	Given the nature of what we have discussed, is there anything else that you would like to comment on or share?	Given the nature of what we have discussed, is there anything else that you would like to comment on or share?

The research team consisted of the PI (KO), a clinician scientist in general internal medicine, a senior scientist with nursing background and experience in qualitative methods (LJ), a pot-doctoral fellow with a background in Industrial Engineering (SHG), and three research analysts. All member of the research team underwent training in qualitative interview techniques at the Li Ka Shing Knowledge Institute at St. Michael’s Hospital and training specific to asking questions surrounding equity and diversity with the ethics committee at Mt. Sinai Hospital.

All interviews were audio-taped and transcribed verbatim then analyzed using directed content analysis [17]. The original interview guide was piloted with three participants. After the first few interviews were completed, the principal investigator (PI) and three members of the research team independently reviewed and used a process of open coding [18] to find themes within the transcripts. At this point, the team met and reviewed each member’s themes. The themes were sorted into categories and the team reached a consensus on an initial coding schema. Following that, an iterative process was used to review the transcripts and derive common themes. Specifically, three iterations of data collection, a combination of open and axial coding [18], and analysis were completed, to enable emerging themes to be explored in more detail in succeeding interviews and ensure saturation of themes. At each iteration, five investigators independently analyzed and coded all the transcripts using the coding scheme. Quotations within the transcripts were highlighted and coded, reviewed by the team, and analyzed to identify links between themes. Three of the five investigators used a process of manual coding and two used Nvivo Pro software [19]. The software was also used to create coding reports and identify links between codes. Inter-rater reliability was achieved using investigator triangulation by cross comparison of the emergent themes for all team members at each iteration [20]. At each step, each case of a difference in coding by any member of the research team was discussed as a group so that all members were comfortable with the code assigned.

As mentioned, caregiver involvement emerged as a critical theme throughout all the transcripts. At the completion of all the iterations of the complete transcripts described above, the team used selective coding [20] within the category of caregiver involvement. Several iterations of coding within the category of caregiver involvement was used to determine areas of focus and derive insights.

## Results

In total, 27 participants completed qualitative interviews. The transcripts from their interviews and discussions while providing their quantitative responses were used for analysis. Most of the interviews were conducted within one week following discharge (21), however, a few were conducted at one month post-discharge (4) or shortly after (2) due to difficulty reaching participants immediately following discharge. Participants who were reached after one week following discharge were asked to reflect on their experiences in the immediate post-discharge period. Although not an inclusion criteria, the participants in this study had an overall high level of caregiver support, with only four participants having no informal support from friends or family, and only one participant with no caregiver support. The majority of interviews were with patients (16) while 40% were with caregivers (11), most of which were adult children (7). Just over half of participants (15) reported high-level of caregiver participation while discharge instructions were being given to the patient (a subjective rating of over 7 on a scale of 0 to 10 by the health care provider giving the instructions). Patients who had high caregiver participation reported slightly higher satisfaction scores (average of 8.2 versus 7.0 on a scale of 0 to 10) and were 40% more likely to have a discussion in hospital on whether they would have the help they needed at home after discharge.

The mean age of patients was 72 years (Table 2). A quarter of participants had a language barrier and close to half had a physical disability impacting their mobility. Over half of the patients lived with their caregivers and relied on them for help with various tasks. The primary caregiver involved in the care for the patient was most often the patient’s family (23); however there was also mention of caregiving provided by friends and the community (4) as well as privately hired professionals (7). The majority of patients (17) had some form of publically-funded home care. In Ontario, Canada, publically-funded home care, often called Community Care Access Centres (CCACs), is available in a limited capacity to patients who require personal care or medical support in the home. Different from caregiving by family or private caregivers, publically-funded services are limited in frequency and duration of services and in the tasks that can be performed. For example, publically-funded caregivers are rarely available to accompany patients to follow up appointments. Participants in our study found that publically-funded support was insufficient, as is illustrated in the following excerpt.

*I can't walk from my bathroom to my bedroom to my kitchen. Now, it is a large condominium, but you know, I can't go into my kitchen and start making meals. So, you know, even with an hour and a half of CCAC, I need more*.

*(patient)*.

**Table 2:** Baseline characteristics of the interview population (n = 27).

	n	%
*Demographics*		
Admission Diagnosis		
Congestive Heart Failure	13	48
Chronic Obstructive Pulmonary Disease	5	19
Pneumonia	9	33
Male Sex	10	37
Age (years) (Mean ± SD)	72 ± 15 (Range 26–95)	
Length of Stay (days) (Mean ± SD)	10 ± 5 (Range 3–18)	
Presence of Language Barrier	6	22
Limited Health Literacy	13	48
Physical Disability impacting mobility	13	48
Sensory Disability	12	44
*Relationship to Caregivers*		
Living with Caregiver	15	56
Reliance on Family for:		
Self-Care	10	37
Food Preparation	11	41
Medication Administration	6	22
Transportation for appointments	12	44
Receive publically funded home care services	17	63

**Fig 1:**
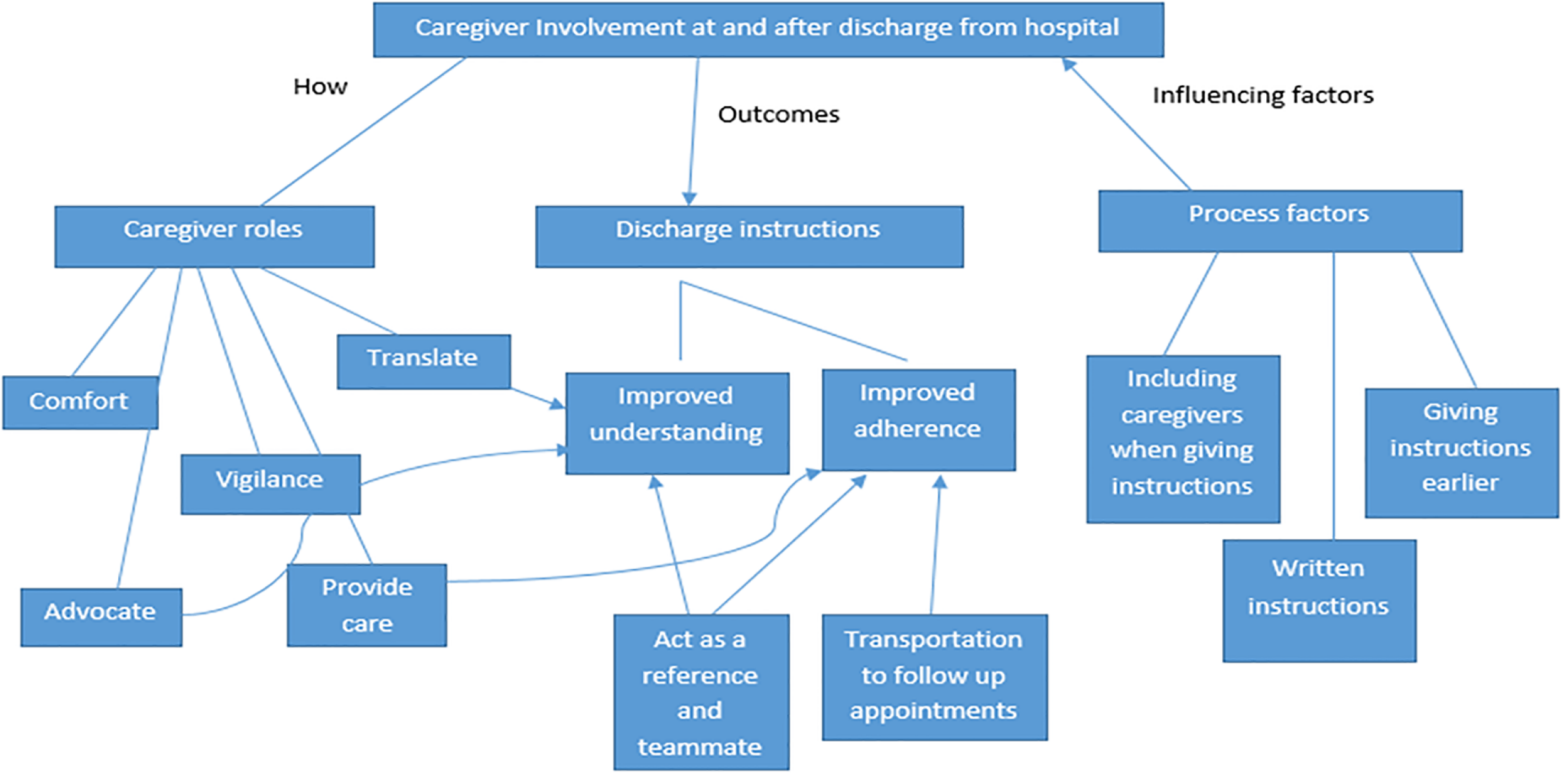
Themes related to caregiver involvement at and after the transition from hospital to home.

Our narrative dataset revealed that caregiving was a critical theme, with almost all participants discussing it in response to the general, open-ended questions asked during the interview. This was true even though patient participants were not directly asked questions related to caregiver involvement. Within the topic of caregiver involvement, there were areas of focus from which we can draw insights (Fig 1): How caregivers are involved after patients are discharged home from hospital, the outcome of having caregiver involvement and support, and influencing factors that impact caregiver involvement when receiving discharge instructions. The remainder of this section presents our findings across these three areas.

### How caregivers are involved

The interviews revealed several caregiver roles after patients are discharged from hospital. Specifically, five subthemes emerged elucidating the integral role caregivers have during care transitions: providing care and assistance, advocating, enacting vigilance, being a source of comfort, and acting as a translator (Table 3). These roles relate to how caregivers are involved in helping patients understand and adhere to instructions, which will also be explained further in the second area of focus.

**Table 3:** Caregiving roles and descriptions.

Role	Description
**Providing care and assistance**	Actively doing tasks for the patient including medical tasks such as wound care and giving medication and daily living tasks such as driving and cooking
**Advocating for patient**	Standing up for the patient to get them what they need or want, such as questioning a doctors decision or requesting additional resources
**Enacting vigilance**	Watching over the patient such as for worrisome signs and symptoms
**Being a source of comfort**	Being with the patient provides a comfortable environment and experience. This also reduces the patient’s anxiety.
**Acting as a translator**	Translating from the patient’s spoken language to English for the health care provider

The predominant subtheme that emerged was providing care and assistance. It reflects how caregivers actively complete tasks for the patient including medical tasks (e.g. administering medication), and thereby assisting the patient in adhering to their discharge instructions, and daily living tasks (e.g. cooking). Unlike publically-funded caregivers, family and other informal caregivers are available to perform these tasks at all hours of the day and night. Some participants expressed how tasks that the patient previously was able to complete independently, had become too difficult after their hospitalization, as illustrated in the following excerpt.

*It’s difficult for me in the night sometimes I have to get up and help him do the suctioning, cleaning the throat… I’m more involved now, because he has to have special attention now because…he can’t do nothing for himself*.

(caregiver)

Another subtheme that emerged was the role of advocacy. The definition of advocacy that emerged through the interviews was that of a person going beyond what is expected to obtain something the patient needs or question the healthcare provider. For example, one caregiver described how she *“asked questions and pressured”* to obtain additional information. Participants expressed how often advocacy was required and the difference it made to their ability to follow their discharge instructions.

Another subtheme that emerged was the role of vigilance. Participants described how caregivers are vigilant by keeping an eye on the patient, watching for worsening symptoms and making sure they are managing okay. For example, one patient expressed that *“I have my family looking after me and that is really important*. *I am lucky*.*”* Patients mentioned how they are in poor physical and mental shape after hospitalization and do not feel able to look after themselves. In several cases, a patient was discharged home on the condition that a caregiver will be there to watch over them, illustrated in the following excerpt.

*All I know is that they asked me the day before if I was going to be here twenty four hours. To keep an eye on her and I said yes. That was one of the conditions for mom coming home*.

(caregiver)

Several interviewees mentioned that caregivers go beyond being a reference for information to being a source of comfort, making patients happy and reducing anxiety. For example, one caregiver described how *“Anytime we [the patient’s children] can help her*, *she is always in a happy mood*.*”* This was especially true for patients with language barriers. They relied on their family to translate for them, often preferring family over a professional interpreter because of the increased level of comfort, as illustrated in the following excerpt.

*I went through all her tests … then I explain what is going on. Because she was kind of nervous too, because there's no translator….she was comfortable when I was telling her what they're doing*.

(caregiver)

### Outcomes of caregiver involvement

The second area of focus that emerged delineated how caregivers helped patients both understand and adhere to their discharge instructions. Many patients were not in a state to understand or remember what was told to them at discharge. Caregivers were often cited as sources of reference. For example, when asked if enough information was given on what to do if they were worried about their condition after discharge, one patient expressed “*I am okay because my sister is a nurse and my sister-in-law has asthma so she is used to some of these medications*. *I am lucky that I have that source of information*. *If I were alone*, *I would be much worse off*.” Other patients were not able to understand their instructions due to language barriers or their cognitive abilities. These patients relied on their caregivers to fully understand their instructions. Participants described the caregiver as a teammate that helps decode instructions, often by advocating by asking the healthcare provider to clarify instructions, and how they take on the task of following discharge instructions together. This is illustrated in the following excerpt.

*My wife and I have been talking about it together and we have gone over the discharge instructions and are maintaining a schedule. She has gone through a lot of the instructions, so she knows what to do. The two of us are doing it together*.

(patient)

The interviews also showed how caregivers increased the patient’s ability to adhere to their discharge instructions. Sometimes, as described in the section above, caregivers actually provide care such as administering medications. For example, one participant described how she needed to break open capsules so her mother would be able to take her medications.

*For some reason when she swallows it’s aggravating. I open up the capsules and put them in something that she can eat it in like a little bit of milk or yogurt*.

(caregiver)

Another component of discharge instructions is follow-up appointments with specialists and primary care physicians. Caregivers had an impact on adherence by booking appointments, providing transportation, and accompanying patients to appointments. In these cases, we see that the patient would not be able to attend their follow-up appointment if not for their caregiver’s involvement.

Patients without informal caregivers had an especially hard time getting to follow-up appointments and often noted poor experiences and despair when discussing their post-discharge experience. This is especially true for patients with mobility impairments, as evident in the excerpt below. When asked about whether she made it to her follow-up appointment, a patient responded:

*Oh honey, I can't walk. I'm tied to an oxygen tank. And I'm not able to use my stairs. I have no one to help me. And I need to take an oxygen tank with me, a wheelchair or a walker. But, I need to have somebody take the wheelchair down in order for me to go anywhere. So, I don't go out of my apartment*.

(patient)

### Influencing factors impacting caregiver involvement

Our interviews revealed a third area of focus around process-related factors affecting caregiver engagement in the discharge process. Specifically, three subthemes illuminated opportunities for caregiver involvement: including caregivers when teaching discharge instructions; providing something clear in writing; and providing instructions earlier in the hospital stay.

Participants appreciated caregivers being included when instructions were provided. Caregivers that were present at discharge were highly engaged and those that weren’t often expressed that they wished they had been. For example, when asked about whether she understood her husband’s medication needs, one caregiver mentioned how “*there was a pharmacist that came in apparently…I wasn’t there for it*,” and therefore she was not clear on which medications were needed.

Participants who spoke about caregivers being included in the discharge teaching seemed to suggest healthcare providers were going the “extra mile” and this strengthened their relationship with the healthcare provider. The following excerpt illustrates this subtheme.

*They kept me apprised of everything. My daughter was always there so they kept her apprised too because she was more alert than I was. So that’s important that somebody else be there and they very much respected that. I think it changed the dynamic with the doctors. Because towards the end if they came in to talk to me and my daughter wasn’t there—they said, ok your daughters not here, and I'd say well she’s coming back in 10 minutes, and then they would come back. Made us much closer*.

(patient)

Participants reported referring to and using written instructions once home. They used them as discussion aids when communicating with healthcare providers in the community. For example, one caregiver said having written instructions allows you to “*Check them out”* referring to taking her written instructions to her community pharmacist and going through the list together. While participants stressed the importance of written instructions, some emphasized it does not replace the role of in-person verbal instructions and dialogue. Overall, participants preferred having additional written instructions. The following excerpt illustrates this subtheme.

*It was explained very well and they gave it to us in writing. He spent time both, I mean Joe and I were both there, he had all written out, he gave Joe a copy, gave me a copy. We looked them all over*.

(caregiver)

This was particularly true for medications, a common source of confusion and frustration experienced by participants, as illustrated by this excerpt:

*I said well listen, can't you just break it down to what she has to take in the morning, what she has to take in the afternoon, and what she has to take in the evening. Put it all down, put it on a notepad, take a photo, a photocopy. Just give me a piece of paper*.

(caregiver)

Another factor impacting caregiver involvement was timing of when discharge instructions were provided. A recurrent theme that emerged was that participants felt discharge was “*too hectic”* and they were unable to focus when instructions were given right at discharge. They felt that providing instructions earlier during the admission would allow more time to ask the required questions to properly understand the instructions, as noted in the following excerpt.

*The discharge is always a bit chaotic. I am afraid that it got a little bit disjointed. I lost track of what I should of been asking. I don't know maybe getting some of the papers the day before you go may help*.

(caregiver)

Caregivers felt that if some instructions were given earlier, if they were given something in writing, and if they were involved in going through the “*information that is on there*,” they could then ask questions when they were there and engaged.

## Discussion

Our study interviewed patients and caregivers in the time following discharge from hospital and identified insights elucidating how caregivers are involved in understanding and adhering to discharge instructions. We also identified process features that, when in place, can enable caregiver involvement. The participants in this study represent an older population with a strong caregiver presence. Over 85% of participants in our study had informal caregiver support and over half lived with their caregivers. However, only 56% of our participants reported high levels of caregiver engagement at discharge. Of the 12 participants who did not report high levels of caregiver engagement, only four did not have an informal caregiver. Therefore, there still remains 30% of patients who had an informal caregiver who was not engaged when discharge instructions were provided. These findings highlight the need as well as opportunities for healthcare providers and institutions to effectively identify targeted ways to involve caregivers during transitions from acute care hospitals.

Caregivers have previously been reported to play an essential role in a patient’s transition home from hospital [21]. Previous studies have shown that informal caregivers facilitate patients’ recovery at home, and improve outcomes including preventing readmissions and death [22, 23]. Specific post-discharge activities caregivers undertake include providing 1) emotional support [24, 25]; 2) medical care, including administration of medications [23, 24]; 3) homemaking and meal preparation services [22, 23]; 4) transport to follow-up appointments [22]; and 5) advocacy and care-coordination [21, 23]. Although there is a growing body of literature around activities caregivers assist with post-discharge from hospital, the role of caregiving has never been highlighted as a factor critical to patient understanding and adherence to discharge instructions. This study identifies caregiving as a critical factor and highlights both the granular and larger roles caregivers play in helping patients with complex medical diseases adhere to discharge instructions. Informal caregivers go through the instructions with the patient, act as sources of reference when patients are not able to act as active recipients at discharge, as well as provide care or complete necessary tasks, and take patients to follow-up appointments. For patients with language barriers or limited health literacy, having caregivers present when receiving discharge instructions also directly impacts understanding. This is especially important in urban centers such as Toronto, Canada, where over 60% of the population has a primary language that is not English [26].

Our study found that patients and caregivers value and want more caregiver involvement. When involved in the discharge, caregivers were more satisfied, evident by higher satisfaction scores and rates of discussions in hospital about whether patients would have the help they needed at home. This is similar to a study by Bull et al. [27] that found caregivers who were more involved in discharge planning had higher satisfaction and were better prepared to provide support to patients. Previous qualitative studies have also demonstrated that caregivers want to play a greater role in improving a patient’s transition from hospital [22, 25, 28–32]. Yet, studies continue to report that caregivers feel unprepared at discharge and are rarely involved in discharge planning [25, 28, 29, 33] highlighting a need for improving how caregivers are engaged when receiving discharge instructions. Previously cited reasons for the lack of caregiver engagement prior to discharge include a lack of dedicated staff time and the growing medical complexity of patients and post-discharge care [34, 35]. As the population ages and patients have more chronic diseases, there are more services to coordinate at discharge, leaving less time for direct patient communication [34, 35], a circumstance intensified by the push for shorter hospital stays [35, 36], which is incentivized in the US and Canada through quality based funding [11, 37]. Some hospitals are now involving transitional care specialists [38], and creating patient-friendly written instructions [13], but few are targeting barriers to engaging caregivers.

Current discharge processes are not optimized to involve caregivers. Our study identified three process factors that impact the ability of caregivers to be involved and, in turn, the impact they are able to have on patients understanding and adhering to instructions: Specifically, involving caregivers during discharge teaching, providing clear written instructions as a reference to verbal instructions, and allowing sufficient time for patient education prior to the day of discharge. Dossa et al. [22] studied communication at discharge and concluded that caregivers should be involved during discharge planning and information should be provided in a legible format. These recommendations echo some of those proposed by participants in this study. A study by Jeffs et al. [39] looked at essential elements of care transition interventions led by nurses and found that successful interventions includes having nurses support patients and caregivers by providing them with education and counseling around self-management post discharge. These previous findings are strengthened and built upon by the recommendations from patients and caregivers in our study.

Strengths of this study are that we looked specifically at the link between caregivers and adherence to discharge instructions, in a cohort of participants with a strong caregiver presence in their lives, and a focus on process factors which present opportunities for action. Existing successful transitional care bundles, such as the care transition intervention [40] and Project RED [41] have elements of these opportunities built into a much larger discharge program. This study pulls these opportunities out as key factors for success specifically related to caregiver involvement.

Limitations of this study are that the interviews were part of a larger, blinded, ongoing mixed-methods randomized controlled trial [15]. In this study, likely half of the participants may have been in the intervention arm and received a discharge instruction sheet which encourages communication of instructions with patients and families at time of discharge, thereby potentially overestimating levels of caregiver engagement. Additionally, six of the 27 interviews were conducted at one-month or longer post-discharge. Although participants were asked to recall the time immediately post-discharge, this may have been difficult to do. However, results of interviews conducted later did not differ from those conducted within one week of discharge. Another limitation was that due to feasibility constraints, participants were contacted by phone, rather than in person, which could have provided additional context to caregiving had they been interviewed and observed in their home setting.

Caregivers clearly play an important part in a patient’s discharge from hospital and during the time immediately post-discharge. Given caregiver impact on understanding and adherence to discharge instructions, it is important for the healthcare team to recognize their role and engage them when reviewing instructions. Understanding the role of caregivers, how they improve understanding and adherence to instructions, and which process factors can improve their involvement is a necessary step for healthcare institutions to consider when developing new transition processes, guidelines, and tools. Future research should study indirect and direct costs of not involving caregivers in the transition from hospital to home.
